# Late onset Li-Fraumeni Syndrome with bilateral breast cancer and other malignancies: case report and review of the literature

**DOI:** 10.1186/1471-2407-12-217

**Published:** 2012-06-06

**Authors:** Karin Kast, Mechthild Krause, Markus Schuler, Katrin Friedrich, Barbara Thamm, Andrea Bier, Wolfgang Distler, Stefan Krüger

**Affiliations:** 1 Klinik und Poliklinik für Frauenheilkunde und Geburtshilfe, Universitätsklinikum Carl Gustav Carus, Fetscherstr, 74, 01307, Dresden, Germany; 2 Klinik und Poliklinik für Strahlentherapie und Radioonkologie, Universitätsklinikum Carl Gustav Carus, Fetscherstr, 74, 01307, Dresden, Germany; 3 Medizinisch Klinik und Poliklinik I, Universitätsklinikum Carl Gustav Carus, Fetscherstr, 74, 01307, Dresden, Germany; 4 Institut für Pathologie, Universitätsklinikum Carl Gustav Carus, Fetscherstr, 74, 01307, Dresden, Germany; 5 MVZ Dr. Reising-Ackermann u, Kollegen, Strümpelstr, 40, 04289, Leipzig, Germany; 6 Gemeinschaftspraxis für Humangenetik, Gutenbergstr, 5, 01307, Dresden, Germany

**Keywords:** Li-Fraumeni-Syndrome, LFS, *TP53*, Secondary cancer, Treatment

## Abstract

**Background:**

Li-Fraumeni-Syndrome (LFS) is an autosomal-dominant, inherited tumour predisposition syndrome associated with heterozygous germline mutations in the *TP53* gene. Patients with LFS are at a high risk to develop early-onset breast cancer and multiple malignancies, among which sarcomas are the most common. A high incidence of childhood tumours and close to 100% penetrance has been described. Knowledge of the genetic status of the *TP53* gene in these patients is critical not only due to the increased risk of malignancies, but also because of the therapeutic implications, since a higher rate of radiation-induced secondary tumours in these patients has been observed.

**Case report:**

We report a patient with LFS harbouring heterozygous, pathogenic *TP53* germline mutation, who was affected by four synchronous malignancies at the age of 40: a myxofibrosarcoma of the right upper arm, bilateral breast cancer and a periadrenal liposarcoma. Radiological treatments and a surveillance program were adjusted according to recommendations for LFS patients.

**Conclusion:**

Management of tumour treatment of patients with LFS is different to the general population because of their risk for secondary cancers in the radiation field. Screening procedures should take a possibly elevated risk for radiation induced cancer into account.

## Background

Li-Fraumeni-Syndrome (LFS; OMIM #151623) is a rare autosomal-dominant, inherited tumour predisposition syndrome associated with an increased risk of a variety of malignancies. Recent statistical analyses stress the relevance of four “core” cancers which account for 77% of all associated cancers: breast cancer, sarcomas, brain tumours and adrenocortical carcinoma (ACC) [1]. LFS is characterized by high penetrance [2] and early-onset tumours [3,4]. The lifetime risk is higher, and age of onset is earlier in women compared to men [5,6]. Several criteria, classical and Li-Fraumeni-like (LFL), have been developed to identify patients and families with LFS [4,7-11] (Table 1). LFS is associated with heterozygous germline mutations in the *tumor protein p53 (TP53)* tumour suppressor gene*.* Mutations in *TP53* are detected in ~80% of individuals fulfilling the classic LFS criteria and ~30% of those fulfilling the LFL criteria (Table 1) [2,12-14]. In order to increase the sensitivity of detection, the Chompret criteria were adjusted in 2009, for age and tumour spectrum parameters (Table 2). Genetic testing for *TP53* is recommended by the National Comprehensive Cancer Network (NCCN, http://www.nccn.org) in accordance with the Chompret criteria, or in any breast cancer patient < 30 years of age testing negative test for *BRCA1* and *BRCA2* mutations.

**Table 1:** Description of different criteria for Li-Fraumeni Syndrome (LFS) or Li-Fraumeni-like (LFL) Syndrome

	**Criteria**	**Description**
LFS classic	Li-Fraumeni [4]	Proband diagnosed with sarcoma before 45 years, AND
		· a first-degree relative with cancer before 45 years, AND
		· another first- or second-degree relative with any cancer diagnosed under the age of 45 years or with sarcoma at any age
LFL	Chompret original [9]	1. Proband with a ”core cancer” before 36 years, AND at least one first- or second-degree relative with
		· cancer (other than breast cancer if the proband has breast cancer) under the age of 46 years OR
		· multiple primaries at any age
		2. Proband with multiple primary tumours, two of which are “core cancers”, with the initial cancer occurring before 36 years, regardless of the family history
		3. Proband with adrenocortical carcinoma at any age of onset, regardless of the family history
LFL	Chompret 2009 update [10]	1. Proband with a tumour belonging to the LFS tumour spectrum before 46 years, AND
		· at least one first- or second-degree relative with cancer (other than breast cancer if the proband has breast cancer) under the age of 56 years OR
		· a relative with multiple primaries at any age
		2. Proband with multiple primary tumours (except multiple breast tumours), two of which belong to the LFS spectrum, with the initial cancer occurring before the age of 46 years, regardless of the family history
		3. Proband with adrenocortical carcinoma or plexus tumour at any age of onset, regardless of the family history
LFL	Birch [6]	Proband with any childhood cancer or sarcoma, brain tumour, or adrenocortical carcinoma diagnosed under 45 years of age, AND
		· a first- or second-degree relative with a typical LFS-related cancer (“core cancers” and leukaemia) diagnosed at any age, AND
		· a first- or second-degree relative in the same genetic lineage with any cancer diagnosed under the age of 60 years
LFL	Eeles [7]	Two different tumours that are “core cancers” or leukaemia in first- or second-degree relatives at any age
Li-Fraumeni Syndrome	NCCN-Guidelines since 2010	Classic LFS-Criteria OR LFL according to Chompret 2001/2009 OR
		Early onset breast cancer: Individual with breast cancer <30 years of age with a negative *BRCA1*/*BRCA2* test, especially if there is a family history of sarcoma, brain tumour, adrenocortical carcinoma or chorid plexus carcinoma

Tumour spectrum: Five “core cancers”: sarcoma, brain tumour, breast cancer or adrenocortical carcinoma plus e.g. leukaemia, lung bronchoalveolar cancer

Abbreviations: NCCN = National Comprehensive Cancer Network.

**Table 2:** Chronologic synopsis of case report

**Year / Month**	**Sarcoma/Carcinoma**	**Stage**	**Therapy**	**Follow up**
2006/08 (40 y)	Myxofibro-sarcoma right upper arm	pT1a, pNx, pMx, G2, R0	Incomplete resection, R1, 2006/08	palpation,
			Re-Resection, R1, 2006/09	12-mo MRI
			Re-Re-Resection with musculo-cutaneous flap 2006/11	
2006/12 (40 y)	Breast cancer right side	ypT2 ypN1a (2/13) yM0 L0 V0 R0 yG3, ER IRS 2, PR IRS 9, Her2neu pos., IRS 3, invasive ductal carcinoma	Neo-adj. CT with 4x FEC, 2007/01-04,	prophylactic
			Lumpectomy and axillary dissection 2007/05,	mastectomy offered
			Adj. CT, 4x P 2007/06-08,	3-mo palpation,
			Herceptin® 2007/09-2008/06	6-mo ultrasound,
			GnRH Analoga + TAM 07/09-ongoing	12-mo MRI
2006/12 (40 y)	Breast cancer left side	ypT1c ypN1mic (1/17) yM0 L0 V0 R0 yG3, ER IRS 8, PR IRS 12, Her2neu pos. IRS 3, invasive ductal carcinoma	see above	see above
2007/05 (40 y)	Liposarcoma periadrenal left	pT2b pN0 (0/2 LK) pMx L0 V0 G3, Rx, pleomorph sarcoma	Lumbal left adrenalectomy 2007/05,	MRI
			Radiation therapy (66 Gy) 2007/08-10	
2008/05 (41 y)	Recurrence Recurrence	ypT2 ypN0 (0/14) ypMx G1 R1(vessel)	CT with 2xIfos 2008/06, followed by Trabectedin, Mini-ICE and Hyperthermia 2009/01-05	
			Compartment resection with hemicolectomy, splenectomy, partial diaphragm resection and resection of 11^th^ costa 2009/06	
2010/06	Recurrence		CT with Trabectedin since 2010/06	

Abbreviations: Neo-adj. = neo-adjuvant, Adj. = adjuvant, CT = Chemotherapy, FEC = 5-FU 500 mg/², Epirubicine 100 mg/m², Cyclophosphamide 500 mg/m², q21d, *P* = Paclitaxel 175 mg/m², q21d, TAM = Tamoxifen®, Ifos = Ifosfamide, mo = monthly, Mini-ICE = Ifosfamid, Carboplatin, Etoposide.

*TP53* encodes a transcription factor implicated in cell-cycle control, apoptosis and genomic stability [15,16]. Impaired TP53 function may not only influence tumour response to radiotherapy and chemotherapy, but also confers an elevated risk for therapy-induced secondary malignancies and possibly increased sensitivity to low-dose radiation exposure by diagnostic methods [4,17].

We hereby present a case with LFS and relatively late tumour onset, in which a *de novo* mutation in *TP53* was identified. Response to adjuvant therapy, treatment modification as well as further screening modalities in patients with LFS are discussed.

## Case presentation

### Myxofibrosarcoma of the right upper arm

In August 2006, a 40-year old female patient was examined for swelling in the lateral side of the right upper arm. Upon resection, the patient was diagnosed with myxofibrosarcoma. A second resection was performed to achieve tumour-free margins and plastic surgery was performed two times on the damaged area for cosmetic purposes.

Screening for metastatic disease by computer tomography (CT) showed no pulmonary, bone or hepatic metastases, however a lesion (36 × 23 mm diameter), classified as a benign tumour by CT criteria, was detected adjacent to the left adrenal gland. A control CT scan in December 2006 revealed that this lesion had increased in size. Biopsy of the periadrenal tumour indicated a possible mesenchymal tumour, however malignancy was not confirmed.

### Bilateral breast cancer

In addition, suspect bilateral mammary lesions were also diagnosed. Histological examination of biopsies taken from both breast tumours, revealed invasive ductal carcinomas on both sides.

Because of an unfavourable breast-tumour-relation, neo-adjuvant chemotherapy was applied. After four cycles of an anthracycline-containing regimen, restaging revealed no significant change in the breast tumours, however progression of the periadrenal mass which extended in diameter to 67 × 49 mm, was identified by CT scan. Firstly, a bilateral breast-conserving tumour extirpation in combination with bilateral axillary lymphonodectomy was performed. Both carcinomas were positive for oestrogen receptor (ER) and progesterone receptor (PR) expression in the majority of tumour cells, and also overexpressed the human epidermal growth factor receptor (HER2/neu) (Table 2). There were no relevant histological signs of regression 3 months following neo-adjuvant chemotherapy.

### Periadrenal liposarcoma

The periadrenal tumour was subsequently resected. Histological diagnosis revealed a poorly differentiated, pleomorphic, periadrenal liposarcoma. The lipogenic nature of tumour was confirmed by immunohistochemical detection of the S100 protein in the tumour cells. Adjuvant chemotherapy with taxanes was recommended following consultation with an interdisciplinary tumour board, because of the nodal positive breast cancer. Post-operative radiation of the periadrenal region due to RX resection of the periadrenal liposarcoma was also scheduled upon completion of the chemotherapy. Treatment with Trastuzumab and endocrine treatment was included according to the receptor status of the bilateral breast cancers. Moreover, radiation therapy after bilateral breast conserving therapy and radiation of the right upper arm was planned.

### Genetic counselling and molecular analysis

Family history revealed a paternal uncle who died at 22 years from a malignancy in the splenic region. No further information regarding this tumour was available. Additionally, the paternal grandfather was diagnosed with leukaemia at 70 years. The maternal grandmother died from colorectal cancer diagnosed at 51 years. Criteria for Li-Fraumeni-like syndrome according to Eeles and the updated Chompret criteria were met (Table 1) [8]. Genetic analysis of *TP53* was performed by sequencing genomic DNA isolated from peripheral blood leukocytes of our patient. A pathogenic, heterozygous germline mutation (p.Arg282Trp) was identified. Neither parent was shown to be a carrier of this mutation. Paternity was confirmed by using 15 high polymorphic Short-Tandem-Repeats. Taken together, these data indicate that the *TP53* mutation in the patient occurred *de novo*, although a germline mosaicism in one of the parents cannot be excluded. We examined tissue from a different germ layer to identify possible somatic mosaicism in the patient. Analysis of DNA from the oral mucosa revealed the same heterozygous *TP53* mutation identified in the leucocytes. Thus, although we cannot rule out somatic mosaicism, this was very unlikely. Predictive testing was performed in the 44 year-old sibling and in both daughters (aged 21 and 18 years) of the patient. All three female subjects were not carriers of the mutation. Analyses of *BRCA1* and *BRCA2* genes in the index patient did not reveal any pathological findings.

### Therapy modification for supposed radiation sensitivity

After confirmation of LFS by molecular analysis, the decision of an interdisciplinary consulting panel was to restrict the planned radiotherapy to the location with the highest priority. This decision was based on the suspected radiosensitivity of individuals harbouring a deleterious mutation in *TP53*. Thus, radiation was scheduled for the periadrenal region with postoperative RX, however the initially intended radiotherapy of both breasts, as well as of the right upper arm was cancelled.

### Surveillance strategies

The patient was offered secondary, bilateral prophylactic mastectomy, to reduce the risk of recurrent breast cancer. Due to the risk of new primary tumours at other sites, the patient refused this option and opted for a close, post-treatment observation regimen. The surveillance program was adjusted to her elevated risk for other primary malignancies and secondary malignancies after radiotherapy. Biannual ultrasound examination of the breast, annual magnetic resonance imaging (MRI) of the breast, and mammography at larger intervals were recommended. Abdominal ultrasound and MRI were predominantly used to monitor the sites of sarcomas.

### Recurrent disease of the periadrenal liposarcoma

In June 2008, multiple nodular lesions were detected close to the left kidney by control MRI, inside and adjacent to the former irradiation region. The optimal response to several lines of treatment with chemotherapy was stable disease. A radical resection of the tumour mass was performed in June 2009. Histological analysis confirmed a recurrent, but well-differentiated liposarcoma. The short interval to radiation therapy of the left periadrenal region indicated resistance of the disease to radiation therapy.

In June 2010, MRI of the abdomen revealed local recurrence. As of February 2012, the patient has been under long-term treatment with trabectedine at a Karnofsky Index of 80%. No distant metastasis, new primaries or recurrences at other sites have since been identified.

## Conclusions

In this report, we describe a 40-year old, female patient with four concurrent primary malignant tumours. The simultaneous occurrence of four malignancies is uncommon, and metastases should be excluded. Indeed, all four tumours exhibited morphological features consistent with their histogenesis. Both breast cancers were hormone receptor positive and included intraductal components as precursor lesions for invasive carcinomas. A recent study by Melhem-Bertrandt *et al.*, described an ER positive/HER2 positive phenotype for breast cancer in *TP53* germline mutation carriers [18]. This study also observed that in cases of bilaterality, both tumours were HER2 positive. In our patient, examination of the entire myxofibrosarcoma did not reveal any lipogenic morphology, and similarly, the liposarcoma did not exhibit a myxoid or non-lipogenic component. Furthermore, the liposarcoma expressed the S100 protein, which is an established marker for lipogenic differentiation. The typical combination of sarcomas and early-onset breast cancer was indicative of LFS and subsequent genetic analysis confirmed a germline mutation in the *TP53* gene. While the patient met some of the wider Li-Fraumeni-like-criteria, she did not meet the classical LFS criteria [4]. The age at onset for the first malignancy was relatively high, as the estimated penetrance at 40 years of age ranges between 77%–100% [5]. The average age for diagnosis of first cancers in *TP53* mutation carriers is estimated to be 21.9 years, and this is earlier in females than in males [1,5]. Predictive testing of minors is recommended since a high prevalence of adrenal gland, soft tissue and brain tumours has been observed in children

Testing of both parents indicated a *de novo TP53* mutation in the patient. *De novo TP53* mutations have been described in up to 23.5% (4 of 17) of carriers [9,19]. Recently, *de novo TP53* mutations were confirmed in 7% and suspected in up to 20% of a larger cohort with 75 germline mutation carriers [20].

### Treatment response to adjuvant therapy and prognosis

Impaired response to chemotherapy and radiation is described in most studies [21-24]. In breast cancer, *TP53* status was identified as independent negative prognostic marker [25], however the results remain controversial. The response to treatment ranges from a high rate of pathologic complete remission of breast cancer after neoadjuvant chemotherapy with anthracyclines, to primary tumour resistance and progression as observed in the case of our patient [26-31]. Introduction of wild-type *TP53* by gene therapy increased the response to chemotherapy or radiation therapy in preclinical and some early clinical trials [27,32-34], however the results were not consistent and to date, gene therapy is not within reach for patients with LFS.

### Treatment-induced secondary cancers

The risk of developing secondary, radiation-induced malignancies was described as elevated in LFS patients, since the first reports of LFS by Li and Fraumeni [3]. Several case-reports point to the appearance of metachronous cancers in radiation-treated areas in cancer patients with *TP53* mutations [35-39].

Data regarding secondary cancers after radiation therapy in young LFS patients is limited, because of the unfavourable prognosis of most core cancers in LFS and the expected time delay in radiation-induced cancers [19]. Interestingly, five long-term in-field relapses or second primary cancers were recently reported in six patients with unilateral breast cancer, following radiation treatment [39]. In a study of 27 LFS patients, nine were treated with radiotherapy. Of these, six patients suffered from one or two successive solid tumours in the radiation field within a period of 3–22 years (median 7 years) after treatment for the first malignancy. One additional cancer was identified in the radiation field of a patient following treatment for a third cancer, after a delay of 7 years [17]. In a separate study, three radiotherapy-treated patients in a series of nine children with adrenocortical tumours and known *TP53* mutations, survived more than two years, however these children developed five secondary malignancies in the radiation fields [19]. Preclinical studies support the hypothesis that cells lacking wild-type TP53 function have an increased likelihood of genetic instability due to high rates of inappropriate recombination after radiation-induced DNA damage (reviewed in Cuddihy *et al.,*[40]). In *Trp53*-heterozygous or *Trp53*-null mice, treatment with low-dose irradiation led to shorter latency of tumour development and a higher incidence of malformations [41-43]. Human *TP53*-deficient cells have been shown to accumulate DNA damage and are susceptible to malignant transformation, whereas *TP53*-competent cells showed cell-cycle arrest facilitating DNA repair, or apoptosis [44]. These data support the observation that LFS patients are more likely to develop radiotherapy-induced secondary cancers. Finally, it appears that in addition to *TP53* variations, the presence of additional genetic alterations are required to predict the individual risk for radiation-induced secondary cancers [45,46].

### Need for therapy modification in LFS patients

Due to the potentially higher cancer induction rate after radiotherapy, it is important to consider the existence of a germline *TP53*-mutation, especially if there is a choice between surgery and radiotherapy [47,48]. After bilateral, breast-conserving resection, the initial plan to apply adjuvant radiation therapy was cancelled due to the detection of a germline *TP53* mutation. Greater effort should be taken in the early detection and complete resection of *TP53*-associated malignancies, since DNA-damaging, standard adjuvant therapies not only have a questionable effect, but also carry the risk for secondary tumours.

### Surveillance

Most of the core cancers of LFS are associated with a poor prognosis. Interestingly, the first prospective data on the successful application of a surveillance programme in *TP53* mutation carriers was recently published. The key imaging procedure was an annual rapid total body MRI starting in childhood. Compared to the control group, this study demonstrated a potential survival benefit using a comprehensive screening program [49].

Genetic counselling and predictive testing should be offered to patients fulfilling the classic LFS or Li-Fraumeni-like criteria, as well as to their relatives, in order to recommend an intensified cancer screening if LFS is confirmed. While the NCCN guidelines recommend the testing of singular cases suggestive for LFS, even in the absence of family history, others negate the necessity for testing because of low mutation rates (0–7%) and high psychological burden. Numerous publications have explored the testing of early onset breast cancer cases for *TP53* germline mutations and found that these represent rare cases [1,11,50-57]. For example, no pathologic mutations were found in 95 patients with breast cancer (< 30 years old), despite the fact that several patients displayed a positive family history for breast and ovarian cancer [50]. As the costs of testing decrease in the future and effective prevention strategies may be confirmed, testing patients with early onset breast cancer without a family history of cancer will become even more feasible.

In comparison to other tumour syndromes, such as hereditary breast and ovarian cancer, prophylactic operations do not offer a good prognosis for carriers of a *TP53* germline mutation*,* and each case should be considered individually. Firstly, the life-time breast cancer risk is estimated to be ~22%. This is significantly lower than for carriers of a pathogenic mutation in *BRCA1* or *BRCA2,* who display a life-time risk for breast cancer of ~60–80%. Secondly, in LFS, malignancies emerge in different anatomical sites, whereas *BRCA*-associated tumours mainly affect the breast and ovaries.

Since some LFS patients seem to carry an elevated risk for radiation-induced malignancies, exposure should be as low as possible, although a general contraindication for any type of radiological diagnostic or treatment cannot be stated. Surveillance strategies should be chosen with regard to the least possible radiation exposure.

## Consent

Written informed consent was obtained from the patient for publication of this case report and any accompanying images.

## Competing interests

The authors declare that they have no competing interests.

## Authors’ contributions

KK has made substantial contributions to conception and design and was writing the manuscript. She made substantial contributions to acquisition, analysis and interpretation of the data. MK revised the manuscript critically for important intellectual content. MS revised the manuscript critically for important intellectual content. KF revised the manuscript critically for important intellectual content and was involved in acquisition and analysis of data. BT was involved in acquisition and analysis of data. AB has made substantial contributions to conception and design and has been involved in drafting the manuscript. She was involved in analysis of data. WD has given final approval of the version to be published. SK has made substantial contributions to conception and design and has been involved in drafting the manuscript. He made substantial contributions to analysis of data. All authors read and approved the final manuscript.

## Pre-publication history

The pre-publication history for this paper can be accessed here:

http://www.biomedcentral.com/1471-2407/12/217/prepub
